# Postnatal Temporal, Spatial and Modality Tuning of Nociceptive Cutaneous Flexion Reflexes in Human Infants

**DOI:** 10.1371/journal.pone.0076470

**Published:** 2013-10-04

**Authors:** Laura Cornelissen, Lorenzo Fabrizi, Deborah Patten, Alan Worley, Judith Meek, Stewart Boyd, Rebeccah Slater, Maria Fitzgerald

**Affiliations:** 1 Department of Neuroscience, Physiology & Pharmacology, University College London, London, United Kingdom; 2 Elizabeth Garrett Anderson Obstetric Wing, University College Hospital, London, United Kingdom; 3 Department of Clinical Neurophysiology, Great Ormond Street Hospital for Children, London, United Kingdom; Boston Children's Hospital and Harvard Medical School, United States of America

## Abstract

Cutaneous flexion reflexes are amongst the first behavioural responses to develop and are essential for the protection and survival of the newborn organism. Despite this, there has been no detailed, quantitative study of their maturation in human neonates. Here we use surface electromyographic (EMG) recording of biceps femoris activity in preterm (<37 weeks gestation, GA) and term (≥37 weeks GA) human infants, less than 14 days old, in response to tactile, punctate and clinically required skin-breaking lance stimulation of the heel. We show that all infants display a robust and long duration flexion reflex (>4 seconds) to a single noxious skin lance which decreases significantly with gestational age. This reflex is not restricted to the stimulated limb: heel lance evokes equal ipsilateral and contralateral reflexes in preterm and term infants. We further show that infant flexion withdrawal reflexes are not always nociceptive specific: in 29% of preterm infants, tactile stimulation evokes EMG activity that is indistinguishable from noxious stimulation. In 40% of term infants, tactile responses are also present but significantly smaller than nociceptive reflexes. Infant flexion reflexes are also evoked by application of calibrated punctate von Frey hairs (vFh), 0.8–17.2 g, to the heel. Von Frey hair thresholds increase significantly with gestational age and the magnitude of vFh evoked reflexes are significantly greater in preterm than term infants. Furthermore flexion reflexes in both groups are sensitized by repeated vFh stimulation. Thus human infant flexion reflexes differ in temporal, modality and spatial characteristics from those in adults. Reflex magnitude and tactile sensitivity decreases and nociceptive specificity and spatial organisation increases with gestational age. Strong, relatively non-specific, reflex sensitivity in early life may be important for driving postnatal activity dependent maturation of targeted spinal cord sensory circuits.

## Introduction

The flexion withdrawal reflex is a fundamental sensory behaviour that protects the body from harm. In adults, it is characterised by a brief ipsilateral contraction of flexor muscles, leading to a brisk, withdrawal of the limb from the source of potential or actual damage on the body surface [1]–[3]. The mature reflex has a high stimulus threshold, normally in the noxious range, and as a result is used extensively in pain research in rodents [4], [5] and in adult man [6], [7]. The flexion reflex reflects the excitability of spinal nociceptive circuits and, as such, has led to much of our current understanding of pain processing including peripheral and central sensitization [8], [9]and descending inhibition [10], [11].

Newborn mammals display clear reflex withdrawal behaviour from noxious stimulation at birth[12] and indeed this reflex has been used to demonstrate hyperalgesia in the presence of tissue injury in human infants and rat pups [13]–[15]. Nevertheless, numerous anatomical and electrophysiological studies in rat pups have shown that the properties of nociceptive reflexes and their underlying dorsal horn nociceptive circuits differ in early postnatal life from those in adults [12], [16]–[18]. Most notably, withdrawal reflexes in rat pups have lower thresholds, in the innocuous range, and are greater in amplitude and duration than adult rats and the underlying dorsal horn neurons and reflex motoneurones have larger, disorganised cutaneous receptive fields [16], [19]–[22]. The changing properties of newborn rodent reflexes are due, at least in part, to the postnatal development of spinal sensory circuits. Newborn rat dorsal horn neurons receive stronger synaptic input from low threshold mechanoreceptor afferents than adults [21], [23] due to exuberant A fibre terminals[17], [24] (Beggs et al., 2002; Granmo et al., 2008) and immature glycinergic signalling [18], [25]. In addition, sensitization of dorsal horn cells [21] and increased fos expression in lamina II [26] are induced by repeated tactile or A fibre stimulation, both of which only occur following noxious stimulation in adults [27].

The implication of these findings for human infants is unclear. While lower cutaneous reflex thresholds have been observed in preterm infants [20], [28], [29], no quantitative analysis has been undertaken. Here, we have performed direct EMG recordings of flexor withdrawal reflex activity evoked by tactile, punctate and (clinically required) noxious stimuli in preterm and full term infants. We show that human flexion reflexes change substantially with gestational age and only gradually become nociceptive specific over postnatal life.

## Materials and Methods

### Participants

Participants were in-patients admitted to the Neonatal Unit and Postnatal ward at University College Hospital (UCH), London, UK. Ethical approval was obtained from the UCH research ethics committee and informed written parental consent given for each infant. For noxious stimulation studies, a clinically required heel lance for blood sampling was performed. No additional heel lances were performed for research purposes.

Table 1 summarises the demographic characteristics of the infants used in this study. Reason for admission included prematurity, respiratory distress or tachypnea, infection, jaundice, and to monitor blood sugar or establish feeds. Infants were divided into two age groups, according to the standard definition of “term” (≥ 37 weeks gestational age at the time of study) and “preterm” infants (<37 weeks gestational age at the time of study). All infants were less than 14 days postnatal age, were neurologically healthy i.e. with no suspected or confirmed clinical neurological disorders or injury and were not prescribed analgesics or sedatives at the time of the study. Infants were not eligible for inclusion in a study if there were signs of tissue damage on the lower limbs, were born with known congenital malformations or genetic conditions or in-utero opioid exposure.

**Table 1:** Demographic characterisation of the neonatal population.

	Age at time of study			
	Preterm		Term	
	<37 weeks GA		≥37 weeks	
	Mean (SD)	Median; range	Mean (SD)	Median; range
Total number in cohort	24		25	
Mean GA at birth, weeks	33.1 (2.2)	33.2; range,28.4–36.0	39.2 (1.7)	38.6; range, 35.9–41.6
Mean GA at time of study, weeks	34.1 (2.0)	34.3; range,30.1–36.4	39.8 (1.7)	39.0; range, 37.0–42.3
Mean postnatal age, days	6.7 (3.7)	6; range, 1–14	3.8 (2.8)	3; range, 0–10
Mean weight at birth, g	1911 (496)	1838; range, 872–2758	3218 (682)	3295; range, 2014–4900
Mean weight at time of study, g	1855 (483)	1825; range, 930–2780	3128 (688)	3285; range, 1969–4900
Number of males	17		12	
Number of multiple gestation births	15		1	
No. caesarean deliveries	22		11	
No. spontaneous vaginal deliveries	2		14	
Condition at birth				
Mean Apgar score @1 min	7.1 (2.2)*	8; range, 5–10*	8.4 (1.6)^+^	9; range, 3–10^+^
Mean Apgar score @5 min	9.2 (0.9)*	9; range, 6–10*	9.4 (1.0)	10; range, 6–10^+^
Mean no. days on mechanical ventilator	1.0 (n = 2)	1; range,1		

SDs are in parentheses. GA, Gestational Age.

* Information unavailable for 2 infants. ^+^Information unavailable for 4 infants.

### Electromyography (EMG)

Flexion withdrawal reflex activity was measured from the lower limb using surface EMG. Self-adhesive bipolar surface silver/silver-chloride electrodes (Cardinal Health, USA) were positioned over the biceps femoris of each limb (including the contralateral muscle where possible). The EMG signal was amplified (x10,000), filtered between 0–500 Hz and sampled at 2 kHz using a 32 bit recording system (Neuroscan Synamps 2, Compumedics, USA). A notch filter was applied at 50 Hz to reduce interference from external electrical signals. A chest electrode served to ground the signal. Surface electrodes were positioned at least 5 minutes prior to cutaneous stimulation to assess EMG recording quality and for baseline data acquisition. EMG recordings were excluded from the data analysis for low signal to noise ratio, that is less than 10% above baseline, or technical artifact.

### Cutaneous sensory stimulation

**Figure 1:**
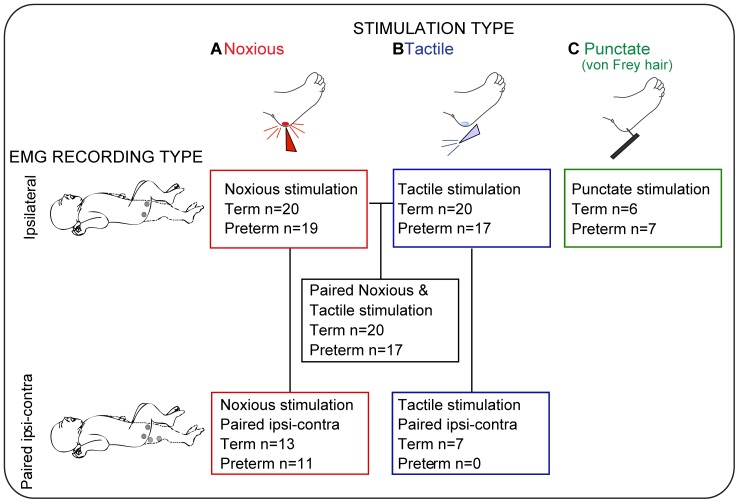
Summary of the experimental groups, stimulation type and EMG recording set-up used in the study. Experimental groups are grouped by stimulation type (A, noxious stimulation – clinically required heel lance; B, tactile stimulation; C, punctate stimulation) and EMG recording set-up (ipsilateral or paired ipsi-contralateral). Schematic of the EMG recording set-up shown on left side of figure panel: ipsilateral (top), or paired ipsi¬contralateral (bottom); grey circles represent surface EMG electrode location over the infant limb.

Figure 1 shows the numbers of infants included for each study. It was not possible to perform all tests on all infants for clinical reasons; noxious heel lancing was only performed as part of standard medical care.

All studies were performed with the participant lying on the side or in the supine position. Three types of stimulus were applied to the foot. (1) Noxious stimulation was a clinically required heel lance performed for blood collection (Tenderfoot; ITC, USA); (Figure 1, inset ‘A’). A lancet device was held against the outer aspect of the heel, a spring-loaded blade was released from the device to break the surface of the skin. (2) Innocuous mechanical “tactile” stimulation was performed in the same group of infants (Figure 1, inset ‘B’) before the lance was applied. Here, the lancet device was rotated by 90° and placed against the heel so that when the blade was released it did not contact the heel nor break the skin. Therefore, infants experienced the tactile sensation and a click associated with the blade release from the lancet device but no skin incision/noxious stimulation. For all infants, the tactile stimulus was performed prior to blood sampling and on the same site on the foot as the noxious heel lance. The time of noxious and tactile stimulation was automatically event-marked on the EMG recording by electronically linking the lancet device to the recording equipment [30] (3) “Punctate” stimuli, performed in a separate study from the lance and tactile stimulation using a series of calibrated von Frey hairs of differing stimulus intensities (Figure 1, inset ‘C’). The von Frey hair threshold was established by applying hairs in ascending order of intensity to the plantar surface of the foot until clear, brisk, leg withdrawal was observed. The observed withdrawal threshold was later confirmed as the threshold that produced an EMG response. The suprathreshold von Frey EMG response was established using a von Frey hair two grades above threshold (mean force applied 6.2±6.0 g, range 0.8 – 17.2 g, at 10 second intervals). Three stimuli were applied and the maximum response used for analysis. To test whether sensitization to repeated punctate stimulation occurred, the EMG response to the threshold von Frey hair was measured before and 30 seconds after a train of 12 suprathreshold stimuli applied at 10 sec intervals (mean ISI, 10.6±0.9 s, range 10.0–12.4 s). Application of each von Frey hair punctate stimulus was event-marked using a custom-built manual push button trigger system linked electronically to the recording equipment.

### EMG Analysis

Raw EMG recordings were filtered with a 10 Hz high-pass filter to eliminate signal drift and ensure signal composition contained frequencies associated with motor activity. Recordings were analyzed from 1000 ms before the stimulus (baseline) to 5000 ms after the stimulus. To identify the onset of the EMG response, the standard deviation was calculated in a 50 ms sliding window from the time of stimulation; the time at which the standard deviation exceeded three times the baseline standard deviation, was defined as the reflex onset. For analysis of the reflex magnitude, the EMG signal was divided into 250 ms time-bins from the onset of the response and the root mean square (RMS) of EMG activity in each time bin calculated. Since the baseline EMG, recorded in the 1000 ms epoch before the noxious stimulus, increased significantly with gestational age [<34 wks: 5.66±3.83 µV (n = 8), 34−<37 wks: 8.10±5.89 µV (n = 11), 37−<40 wks: 11.53±4.05 µV (n = 10), <43 wks: 17.85±4.30 µV (n = 10); One-way ANOVA, **p = 0.003], evoked EMG activity was expressed as fold changes over baseline. Data points with fold changes greater than 100 were excluded as artifact.

### Statistical analysis

Statistical analyses were conducted using GraphPad Prism (GraphPad Software, USA). Data expressed as mean (standard error of the mean, SEM), unless otherwise stated. All groups of data were tested for normality using the D′Agostino-Pearson normality test, where the data did not follow a Gaussian distribution an appropriate non-parametric test was applied (specified in the text parenthesis). Differences in the patterns of EMG activity were compared for (1) gestational age –preterm versus full term infants, (2) laterality – ipsilateral versus contralateral biceps femoris activity, and (3) stimulus specificity – non-noxious tactile stimulus versus noxious stimulus, using a two-way ANOVA with repeated measures. Posthoc analyses of these comparisons were conducted using appropriate t-tests (specified in the text parenthesis). Significant differences were assumed at p<0.05. T-tests are reported with degrees of freedom, t-statistic and significance level. ANOVAs are reported with degrees of freedom (between groups, within group), F-statistic and significance level. Asterisks indicate the significance level where: *, p<0.05; **p<0.01; ***p<0.001.

## Results

### Nociceptive flexion reflexes are greater in magnitude and duration in younger infants

**Figure 2:**
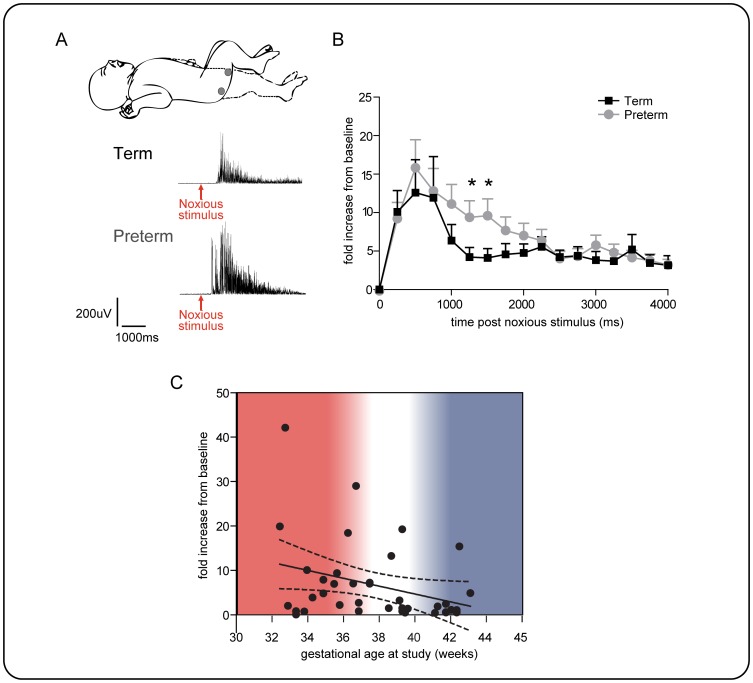
Nociceptive flexion withdrawal reflex activity in preterm and term infants. A: Experiment set-up, surface EMG leads were placed over the ipsilateral biceps femoris to record flexion withdrawal reflex EMG activity evoked by a heel lance, example trace from term and preterm infant below; arrow indicates time of noxious stimulus. B –comparison of flexion reflex biceps femoris EMG activity evoked by a heel lance in term (black, n = 20) and preterm infants (grey, n = 19). Data is expressed as fold increase in EMG activity measured in 250 ms time epochs for 4 seconds post-stimulus. The two groups are significantly different [two-way ANOVA, *p = 0.04 age group, ***p = 0.001 time]. Significantly greater responses were recorded in the 1000–1500 ms post-stimulus epochs in preterm infants [Student's unpaired t-test, 1000–1250 ms, *p = 0.04, 1250–1500 ms, *p = 0.04]. C: A graph showing correlation between EMG amplitude in the 1000–1250 ms epoch after heel lance and gestational age at study (R2 = 0.1; P = 0.025, n = 39).

To test whether infant nociceptive flexion reflex responses change with gestational age, we compared the biceps femoris EMG activity evoked by a clinically required time-locked heel lance in preterm (<37 weeks gestation at time of study, n = 19) and term infants (≥ 37 weeks gestation at time of study, n = 20). In all infants, a robust biceps femoris EMG response was evoked by heel lance, but the overall nociceptive flexion reflex response was significantly greater in preterm compared to term infants due to more sustained EMG activity in the younger group (Figure 2).

Figure 2B shows that while the lance evoked EMG activity in both groups peaks within the first 1000 ms, the magnitude and time course of activity is significantly different in preterm compared to term infants [two-way ANOVA: age group, F1,629 = 5.3,*p = 0.04; and time course of activity, F16,629 = 5.9, ***p = 0.0001 time]. The nociceptive reflex activity in the 1000–1500 ms time period post stimulus is significantly greater in the younger babies (Welch-corrected unpaired t-test, 1000–1250 ms, t24 = 1.8, *p = 0.04; 1250–1500 ms, t24 = 1.7 *p = 0.048). Furthermore, Fig 1C shows that lance evoked EMG activity in individual infants at 1000–1250 ms post-lance is correlated with gestational age at study (R^2^ = 0.1, *p = 0.025, one-tailed). Taken together, this data shows that cutaneous noxious evoked flexion reflex EMG activity decreases with gestational age in human infants.

### Noxious stimulation evokes equal ipsilateral and contralateral flexion reflexes in newborn infants

**Figure 3:**
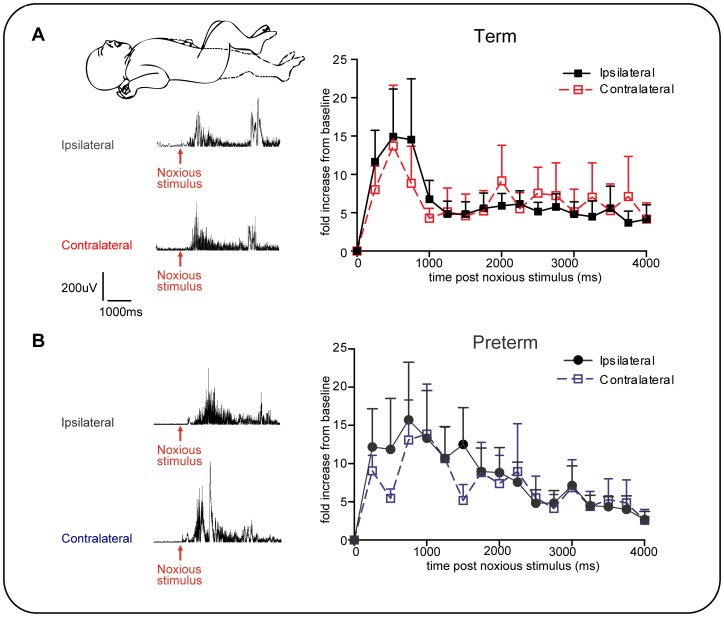
Noxious stimulation evokes ipsilateral and contralateral flexion activity in preterm and term infants. A: Evoked activity on the two sides are not significantly different in A, term infants (n = 13) and B: preterm infants (n = 11). Inset: Experiment set-up, surface EMG leads were placed over the ipsilateral (filled lines) and contralateral (dashed lines) biceps femoris to record flexion reflex EMG activity evoked by a heel lance. Example trace from term and preterm infant below, arrow indicates time of noxious stimulus.

To test the spatial characteristics of human infant nociceptive reflexes, we performed paired EMG recordings from the ipsilateral and contralateral biceps femoris following noxious heel lance (preterm, n = 11; term n = 13). In both preterm and term infants, noxious stimulation of the heel evoked EMG activity in the contralateral limb that was not significantly different in magnitude and duration from the ipsilateral flexor reflex EMG activity (Figures 3A & B). This data shows that in both preterm and term infants, nociceptive flexion reflexes are not restricted to the stimulated limb.

### Flexion reflexes are not always noxious specific in infants

To test whether human infant flexion reflexes are nociceptive specific, the reflex response to a non-noxious tactile stimulus was recorded (where the lancet device was rotated by 90° and placed against the heel so that when the blade was released it did not contact the heel nor break the skin) and compared to the response to a heel lance, in the same infants. In both age groups, some, but not all, infants displayed a significant flexion reflex response to innocuous tactile stimulus. The occurrence of these responses to non-noxious, tactile stimulation of the heel did not differ in the two gestational age groups [term: 40% (n = 8/20); preterm 29% (n = 5/17) respond to tactile stimulation]. Furthermore, there was no significant difference in the magnitude or duration of the noxious evoked EMG response in those infants that displayed a tactile response (‘tactile responders’ – infants who responded to tactile stimulation with a brisk leg withdrawal) and those that did not (‘tactile non-responders’ – infants who did not respond to tactile stimulation with a brisk leg withdrawal), (term infants: two way ANOVA, tactile sensitivity, F1,288 = 0.2, p = 0.65; time course of activity. F15,288 = 1.7, p = 0.06).

**Figure 4:**
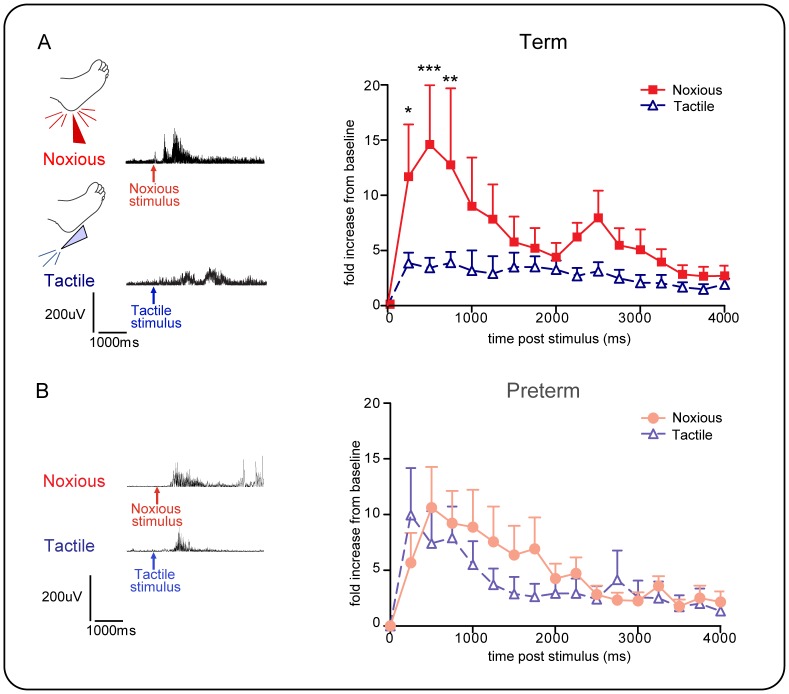
Flexion withdrawal reflex biceps femoris EMG activity evoked by noxious and tactile stimulation. Only data from those infants that displayed a paired tactile and lance response is shown here. A. term (n = 7) and B. preterm infants (n = 5). Data is expressed as fold increase in EMG activity measured in 250 ms time epochs for 4 secs post stimulus. While in term infants (A) the flexor reflex response to noxious lance stimulation is significantly greater than to tactile stimulation, in preterm infants, the two responses are not significantly different. [Term infants two-way ANOVA, **p = 0.006 time, ****p<0.0001 stimulus. Student's t-test, at 250 ms, *p = 0.014, at 500 ms, ***p = 0.0005, and at 750 ms, **p = 0.006].

Fig.4 shows paired comparisons of noxious and tactile flexion reflexes in ‘tactile responders’. Figure 4A shows that, in term infants (n = 7), the tactile evoked EMG response was significantly smaller than the noxious evoked EMG response, but Figure 4B shows that in preterm infants (n = 5), the magnitude and duration of evoked response to tactile stimulation was not significantly different from that evoked by noxious stimulation (Term infants, two-way ANOVA, **p = 0.006 time, ****p<0.0001 stimulus. Fisher's t-test at 250 ms: *p = 0.014, at 500 ms: ***p = 0.0005, at 750 ms: **p = 0.006). Furthermore, tactile stimulation of the heel evoked both ipsilateral and contralateral reflexes, but contralateral reflexes were significantly smaller in magnitude than ipsilateral responses [Term infants, two way ANOVA: side, F1,64 = 6.1, *p = 0.01; time course of activity, F15,322 = 2.0, *p = 0.01, n = 7; no recordings for preterm infants].

These results show that flexion reflex EMG activity is not necessarily modality specific in newborn infants and can be evoked by non-noxious tactile stimulation. Importantly, in preterm infants, tactile reflexes, when they occur, are not significantly different from noxious lance evoked responses, whereas in term infants noxious evoked reflexes are significantly larger than tactile evoked reflexes.

### Infant flexion reflexes are sensitized by brief, low frequency punctate stimulation

**Figure 5:**
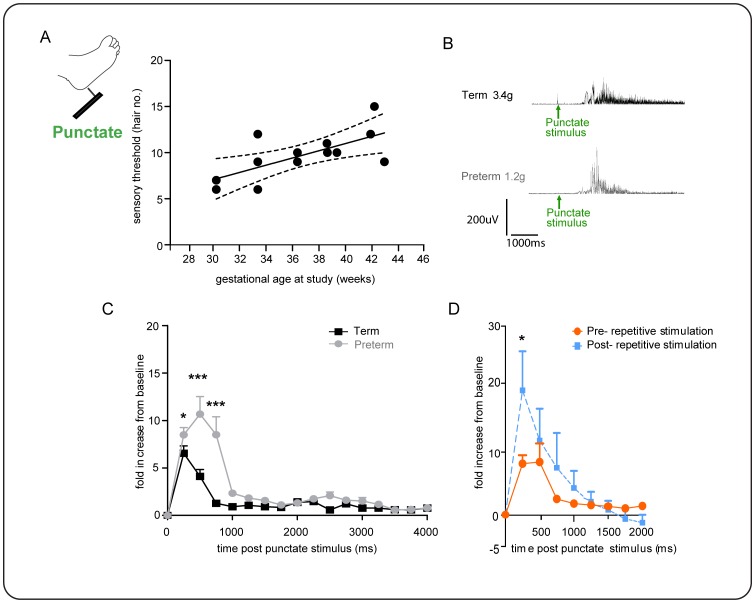
Single and repeated punctate stimulation evokes flexion activity that is gestational age dependent. A, Cutaneous sensory threshold to punctate stimulation significantly increases with gestational age; B, threshold force required to evoke leg withdrawal and example flexion reflex EMG traces from term and preterm infants are shown. C, Threshold flexion reflex EMG responses in preterm infants are significantly greater than in term (two-way ANOVA, ***p<0.0001 age group, **p = <0.0001 time; Fisher's unpaired t-test, 250 ms, *p = 0.02; 500 ms, ***p<0.0001, 750 ms, ***p<0.0001). Note- two EMG recordings excluded from the analysis due to technical artefact. D, Brief, low frequency repeated punctate stimulation sensitised infant flexion reflex EMG activity as shown by a significantly larger flexion response to a threshold hair after 10 seconds of repeated stimulation, compared to the responses to the same threshold hair when tested 10 s before the train (two-way ANOVA, *p = 0.04 treatment,***p = 0.002, time; Fisher's paired t-test, 250 ms, *p = 0,002, n = 11, pooled term and preterm.

To explore the cutaneous sensitivity of infant flexion reflexes further, we applied calibrated punctate von Frey hairs to the foot (vFh) in a separate group of infants (n = 11, Fig.5). Application of a known force to the skin surface allowed precise measurements of cutaneous reflex thresholds and reflex excitability. Von Frey thresholds were initially recorded using visual observation of leg movement and confirmed from post hoc EMG analysis. Fig 5A shows that the von Frey threshold of infant flexor reflexes significantly increase with gestational age (R^2^ 0.46, **p = 0.008, one-tailed). The median (IQR) threshold in preterm infants was 1.23 (0.43–2.75 g, n = 6) and in term infants was 2.1 g (1.67–3.88 g, n = 5). In addition, the magnitude of flexion EMG responses to suprathreshold vFh punctate stimulation (2 grades above threshold) is significant greater in amplitude and duration in preterm infants (n = 6) compared to term infants (n = 5) (Two-way ANOVA, age, F1,154 = 7.4, **p<0.007; time course of activity, F16,154 =  5.3, ***p = <0.0001; Fisher's unpaired t-test, 250 ms, *p = 0.02; 500 ms, ***p<0.0001, 750 ms, ***p<0.0001).

We also tested whether von Frey hair evoked flexion reflexes could be sensitized by repeated stimulation. To do this a suprathreshold hair (2 grades above threshold) was applied to the foot repeatedly at a rate of 1/10 s for two minutes (n = 6 preterm, n = 5 term). A reflex response was evoked an average of 5 times by these 12 repeated stimuli in both age groups. Figure 5E shows that 10 seconds after repeated stimulation, the reflex EMG response to a threshold hair was significantly larger than the response to the same threshold hair when tested 10 seconds before the train (Two-way ANOVA, *p = 0.04 treatment,***p = 0.002, time; Fisher's paired t-test, 250 ms, *p = 0,002, n = 11, pooled term and preterm, first 1000 ms).

These results show that the flexion reflex in preterm infants is more sensitive to cutaneous punctate von Frey hair stimulation than term infants and that infant flexion reflexes are sensitized by repeated innocuous punctate stimulation.

## Discussion

These results demonstrate that human infant spinal nociceptive reflexes undergo substantial changes in modality, temporal and spatial specificity with gestational age. In contrast to adults, flexion reflex EMG responses to cutaneous noxious stimulation are remarkably prolonged and widespread, and display marked sensitivity to single and repeated non-noxious tactile and punctate stimulation. These data provide new insight into the functional maturation of neural pathways underlying early human pain behaviour.

### Differences in adult and neonatal nociceptive reflexes in man

In adult man, the lower limb flexion reflex is a brief response that results in limb withdrawal from a noxious or potentially noxious cutaneous stimulus and is a reliable and objective reflection of an individual's pain experience [6]. Stimulation of small diameter nociceptive fibres is required to produce this relatively short duration reflex in healthy adults [31], [32]. Thus high intensity cutaneous nerve stimulation in adults produces a distinct burst in biceps femoris, which is linearly correlated with pain sensation and returns to baseline 100–120 ms poststimulus [33]. Thermal laser stimulation of the foot also elicits a very brief (∼100 ms), long latency nociceptive reflex that is well correlated with pricking pain sensation [34].

In contrast, we show here that the infant flexion reflex is remarkably long duration. A single heel lance evoked biceps femoris activity for at least 2–4 seconds, decreasing significantly between the preterm and term period, but remaining longer in duration than adult reflexes. Furthermore, the reflex does not always require a noxious stimulus in infants, but can also be evoked by tactile and punctate innocuous skin stimulation. Newborn infants are known to have low cutaneous thresholds [29], [35] but here we show, using within infant comparisons, that the reflex response to touch can be as great as that to noxious stimulation in the youngest babies. In addition, the infant contralateral flexion response to noxious stimulation is as large as the ipsilateral response, while in adults it is entirely ipsilateral [36]. Finally, repeated punctate innocuous stimulation in infants caused a significant increase in reflex magnitude and a drop in threshold, which are the hallmarks of sensitization [28]. In contrast, flexion reflex temporal summation or sensitization occurs in adults only after repeated C fibre or noxious stimulation [37], [38] and is associated with increased pain ratings [39].

### The neural basis for infant human nociceptive reflex properties

The results reported here have a clear translational link with laboratory animal data. The first seven days in rodent life approximates to the preterm period in man from 24 weeks gestation to term equivalent. Rat pups show a postnatal decline in the amplitude and duration of flexion reflex response to a single strong mechanical skin stimulus [40]; at birth the reflex response in rats can last for up to 20 seconds but by the third postnatal week, even the highest intensity stimulation never produces reflexes of that duration. This pattern is likely to reflect lack of inhibitory control in cutaneous, rather than motor, spinal circuits [12] since it is not reflected in proprioceptive reflexes [41], [42]. While the excitatory and inhibitory locomotor circuitry in newborn rats closely mimics that of adults [43], cutaneous reflex receptive fields are large and disorganised at birth and take several weeks to mature [16], [22]. Neonatal dorsal horn cells also have larger receptive fields and display prolonged firing to noxious stimuli [19], [44]. Furthermore, the balance of descending control from the brainstem onto neonatal nociceptive dorsal horn circuits is excitatory rather than inhibitory which also enhances reflex sensitivity [45]. Repetitive activation of low-threshold A-fibres sensitizes neonatal dorsal horn neurons [21], evokes fos expression[26]and NMDA dependent LTP [46] in the dorsal horn all of which require high threshold stimulation in adult animals. There is evidence for a greater synaptic input from cutaneous A fibre myelinated afferents in the newborn rat [23], [47]–[49], due to diffuse A fibre terminal fields [17], [24]and immature inhibitory signaling, in particular from glycinergic networks, in the dorsal horn[25]. In adults, Aβ fibres synapse directly onto glycinergic interneurons [50], [51]and glycine receptor antagonists enhance tactile behavioural sensitivity [52], [53]and increase A fibre excitation of dorsal horn neurons [54], [55]. However in the newborn cord, glycinergic synaptic activity is weak [56] and for the first two postnatal weeks, glycine facilitates rather than inhibits dorsal horn wide dynamic range neuronal responses to low threshold skin stimulation [25].

### Developmental implications of nociceptive reflex immaturity in healthy neonates

The developing nervous system depends upon sensory input for tuning and organising sensory and motor circuits and we propose that the sensitivity of newborn reflexes to low threshold skin stimulation reflects this need. The newborn mammal is almost continuously exposed to tactile skin stimulation, be it through maternal contact, huddling or spontaneous twitching [16]. Maternal licking and grooming during a critical period in early life modifies neural development in newborn rat pups [57]and in human infants, there is evidence that skin contact supports normal growth and development [58]. The maturation of directed nociceptive reflex behaviour between the second and third postnatal week, requires intact low threshold inputs [16] and the postnatal organisation of primary afferent terminals in the dorsal horn is prevented if animals are raised in a high ‘tactile noise’ environment [17]. The first postnatal weeks in rodents, when many spinal sensory synaptic and circuit changes take place, are equivalent to infancy and early childhood in man [59]. Thus we propose that the cutaneous sensitivity of human infant reflexes reported here will maximise sensory drive at critical stage of somatosensory and pain circuit development [18].

### Do flexion reflexes reflect pain perception in neonates?

The implications of these findings are considerable for understanding human somatosensory development and for clinical pain assessment of neonates. In adults nociceptive flexion reflexes are a useful measure of central pain processing: they are sensitized by repeated C fibre stimulation [38] enhanced in chronic pain states [60], [61], modulated by distraction [62] and emotional arousal [63], and reflect ethnic differences in pain report [64]. The size of the reflex receptive field area in adults can be modulated by the cognitive state demonstrating a link between the cognitive activity and the descending control of spinal withdrawal reflex pathways [65] and long-term potentiation of the flexion reflex has been proposed as a target for pain therapy [66]. The correlation of flexion reflex threshold and magnitude with subjective pain threshold and intensity in adults, suggests that such a link may also apply in infants. The immaturity of the infant brain makes the concept of subjective pain experience merely speculative, but it is possible to draw comparisons between pain processing at the spinal and cortical levels in preterm and term infants. Thus the sensitivity of the preterm flexion reflex to tactile inputs is entirely consistent with the lack of differentiation between tactile and noxious cortical activity before 35 weeks gestation [67]. It also agrees with reports that the behavioural and physiological reactivity of preterm neonates to tactile stimulation can be the same as that to painful stimulation [68], [69]. Caution should therefore be used when using behavioural indices as a measure of pain in infancy. Our results confirm the importance of quantitative measurement of the neural activity underlying early human tactile and pain behaviour.
